# Oral itraconazole for the treatment of giant tufted angioma with hair loss arising during pregnancy: A case report

**DOI:** 10.1111/1346-8138.15144

**Published:** 2019-11-14

**Authors:** Wei Wu, Jianqiang Shi, Zhaojun Li, Ding Li, Shuhui Dou, Sushmita Pradhan, Xin Ran, Yuping Ran

**Affiliations:** ^1^ Department of Dermatovenereology Affiliated Hospital of Guangdong Medical University Zhanjiang China; ^2^ Department of Dermatovenereology West China Hospital Sichuan University Chengdu China

Dear Editor,

Tufted angioma or Nakagawa angioblastoma is a benign angiogenic neoplasm of pediatric populations rarely reported in adulthood and pregnancy.^1^, ^2^ We report a case of huge tufted angioma arising during pregnancy with severe pain and hair loss, resulting in the improvement of the symptoms and hair regrowth by oral itraconazole.

A 35‐year‐old Chinese woman 1 month after her third childbirth presented with progressive papules and plaques on the nape and occiput, accompanied by severe pain for 3 months. She started developing papules and plaques at the nape during her 8‐month pregnancy, which proliferated and extended to the occiput with hair loss and severe pain. She vaginally delivered a healthy girl. The lesions and pain persisted after delivery. She reported no drug history during gestation and post‐partum. Physical examination revealed a cluster of fuscous infiltrating plaques and nodules sized approximately 4 cm × 10 cm, annularly and unevenly distributed over the nape and occiput with hair loss (Fig. 1a). Histopathology showed a “cannon ball‐like” appearance (Fig. 1b), demonstrating many scattered, clearly limited, round cellular lobules in the dermis, and subcutaneous area composed of hyperplastic vascular endothelial cells and perivascular cells surrounding dilated lymphatic vessels (Fig. 1c). Immunohistochemistry revealed positive capillary aggregates for CD31 (Fig. 1d), CD34 (Fig. 1e), D2‐40 (Fig. 1f) and positive perivascular cells for smooth muscle actin (Fig. 1g), but was negative for desmin and S100 protein. Diagnosis of tufted angioma was confirmed.

**Figure 1:**
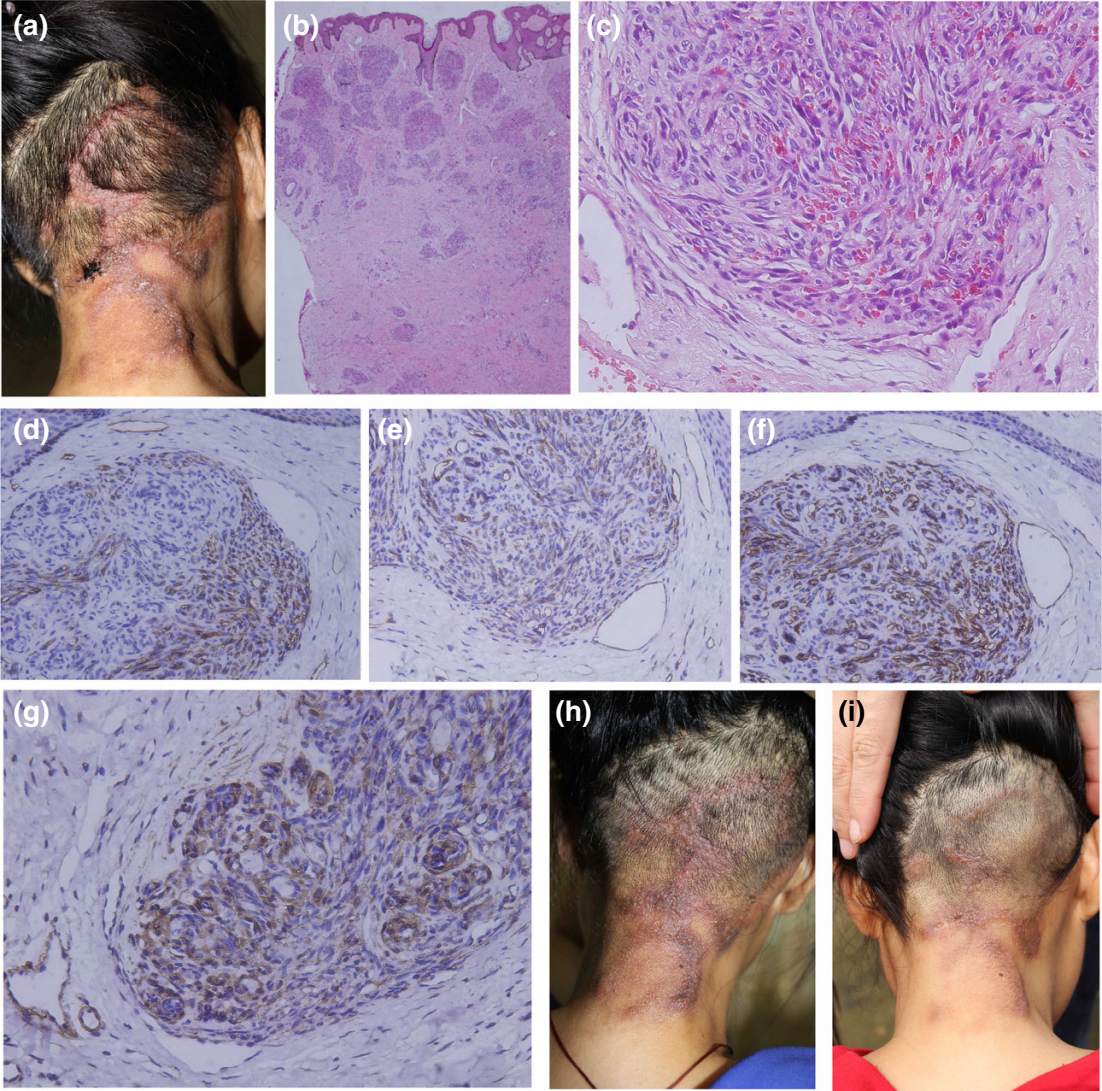
(a) Lesions prior to treatment. (b) Histopathology showing a “cannon ball‐like” appearance (hematoxylin–eosin [HE], original magnification ×20). (c) Vascular tufts composed of hyperplastic vascular endothelial and perivascular cells (HE, ×200). Immunohistochemistry showing the spindle tumor cells positive for (d) CD31 and (e) CD34 (×200). (f) Lymphatic vessels were positive for D2‐40 (×200). (g) Perivascular cells were positive for smooth muscle actin (×200). (h) The lesions improved after 2 weeks of oral itraconazole. (i) Sustained clinical improvement with hair regrowth after 6 months of itraconazole withdrawal was achieved.

After detailed explanation and signed informed consent was obtained, itraconazole capsules (Xi’an Janssen Pharmaceutical, Xi’an, China) 200 mg twice daily and quitting of breast‐feeding was initiated. The pain was significantly relieved after 3 days. Two weeks later, the plaques slightly flattened (Fig. 1h) and the pain disappeared. Hair regrowth was observed after 1 month. Itraconazole was discontinued after 3 months. Significant portions of the lesions disappeared. Liver function and blood routine were normal before and after the treatment. After 6 months of itraconazole withdrawal, sustained clinical improvement (Fig. 1i) was observed.

The pathogenesis of tufted angioma is unclear and may be associated with some vascular growth factors. Pregnancy could be a predisposing factor for vascular proliferation, indicating estrogen promoting its development.^1^, ^2^ Tufted angioma may occur in hypertrichosis and hyperhidrosis;^2^ however, hair loss was never reported. It was regretful that the histopathology of hair loss was not collected. Complete surgical excision is recommended for a small lesion. Topical rapamycin, cryotherapy, electron beam radiation and pulsed dye laser are also applied. Systemic administration includes corticosteroid, aspirin, propranolol, interferon (IFN)‐α, rapamycin and vincristine. Ran *et al.* 3, 4 first reported successful treatment of oral itraconazole with 5 mg/kg per day for infantile hemangiomas, and found that itraconazole significantly reduced platelet‐derived growth factor (PDGF)‐D level, resulting in suppression of PDGF receptor‐β activation, and inhibition of its downstream effectors, such as PI3K, Akt, 4E‐BP1 and p70S6K.^5^ However, the therapeutic effect of itraconazole for tufted angioma is still unclear. Itraconazole is an antifungal drug with good tolerance, unlike corticosteroid, propranolol and IFN‐α which have side‐effects, and rapamycin being an infrequent drug. Therefore, itraconazole can be used for the treatment of large areas and painful tufted angioma.

## Conflict of Interest

None declared.
